# Assessing the Unmet Social Needs and Financial Hardship of Cancer Caregivers: A Scoping Review

**DOI:** 10.1007/s11912-025-01726-7

**Published:** 2025-10-22

**Authors:** Bridgette Thom, Stephany Morales Melendez, Greg Seltzer, Brianna Connelly, Tess Thompson

**Affiliations:** 1 School of Social Work, University of North Carolina (UNC), Chapel Hill, NC USA; 2 UNC Lineberger Comprehensive Cancer Center, Chapel Hill, NC USA

**Keywords:** Social needs, Oncology, Caregiver, Financial hardship, Health-related social needs

## Abstract

**Purpose of Review:**

The objective of this scoping review was to characterize published literature on unmet social needs and financial hardship among cancer caregivers. We sought to describe study attributes; assess the associations of needs with physical and psychosocial outcomes; identify tools to measure caregiver social needs and financial hardship; and examine needs-related interventions for caregivers.

**Recent Findings:**

Thirty-five articles were included in the review, most of which (60%) were published in the past three years (2022–2024). Financial hardship was addressed in nearly all of the included studies. There was a range of measurement tools used across the studies, and 37% of studies assessed social needs/financial hardship as part of a measure of caregiver burden. Most studies (92%) were cross-sectional and did not include a theoretical framework (59%), with only one study describing a caregiver-focused intervention. Most qualitative studies (*n* = 4/5) captured the interconnection among social needs.

**Summary:**

There is limited exploration of unmet social needs and financial hardship among cancer caregivers. Review findings demonstrate the need for increased research, both descriptive and interventional, on the social needs and financial hardship of cancer caregivers.

## Introduction

Social determinants of health, such as education and income, have a well-established association with access to oncology care, adherence to provider recommendations, and overall outcomes [1]. In recent years, a growing body of work has focused on measuring and addressing unmet social needs in patients with cancer [2]. These social needs are the individual-level manifestations of upstream conditions and include factors such as food insecurity, transportation difficulties, and economic hardship [3, 4]. In a prior scoping review, we explored the reporting and measurement of social needs among patients with cancer and cancer survivors, finding considerable variation in measurement, robust attention to patients’ financial concerns, and associations between unmet social needs and lower quality of life [1]. 

Informal caregivers, who provide crucial emotional, tangible, and informational support to patients and survivors, often face negative financial impacts due to their caregiving responsibilities and may have unmet social needs of their own. A well-established body of literature has addressed the complexity of cancer caregiving, including the concept of caregiver burden and associated interventions, experiences of distress and bereavement among caregivers, and the social support needs of caregivers [5–9]. Previous analyses have characterized the expenses associated with cancer caregiving, with estimates ranging from $2877–$4806 per month, and a recent review highlighted the interaction of financial hardship with the social and legal needs of patient-caregiver dyad [10, 11]. The Dyadic Cancer Outcomes Framework depicts how the dyadic interaction between caregivers and patients during the cancer care trajectory may be informed by their social context, including broader social determinants of health and individual-level social needs (both those of the patient and of the caregiver) [12]. Addressing the social needs and financial concerns of caregivers thus has the potential to impact outcomes for the patient, the caregiver, and the relationship between the patient and caregiver.

The objective of this scoping review was to characterize published literature on unmet social needs and financial hardship among caregivers of patients and survivors with cancer. Specifically, we sought to describe study and sample attributes; assess the associations of caregiver unmet social needs with physical and psychosocial outcomes; identify tools to measure and assess unmet social needs and financial hardship in caregivers; and examine needs-related interventions for caregivers.

## Methods

### Search Strategy

Following the Preferred Reporting Items for Systematic reviews and Meta-Analyses extension for Scoping Reviews (PRISMA-ScR), we conducted a scoping review to characterize the current literature related to the social needs of cancer caregivers [13]. As in our prior work, we limited the review to articles addressing at least one potentially modifiable individual-level social needs commonly included on screening tools: food insecurity, financial/economic hardship, utility assistance, employment difficulty, housing or transportation concerns, or personal safety [1]. 

Search terms were drawn from our previous scoping review on the social needs of cancer survivors and were adapted to include terms relating to caregiving, including “caregiver” and “care partner.”^1^ The search was conducted in November 2024 across 6 databases (APA PsycInfo, CINAHL, Embase, Medline/Pubmed, Web of Science), and two members of the research team (BT, TT) reviewed the reference lists of relevant articles to identify additional potential articles to include.

### Inclusion/exclusion Criteria

Articles were included in the review if they met the following criteria: (1) peer-reviewed journal articles published in English from 2000 to 2024; (2) original empirical data (qualitative or quantitative) from a sample of cancer caregivers; (3) inclusion of caregivers of U.S.-based samples of patients with cancer or cancer survivors diagnosed with cancer as adults (i.e., not pediatric patients); (4) assessment of financial hardship or at least one social need noted above.

Articles were excluded as follows: (1) included only demographic data without a specific assessment of a social need as an independent or dependent variable; (2) included only caregivers of patients with non-melanoma skin cancer; (3) included only patient/survivor data despite mentioning caregivers; (4) combined patient/survivor responses with caregivers responses such that caregiver responses were indistinguishable; (5) was a dissertation, case report, protocol paper, conceptual paper, commentary, editorial, or published abstract; (6) was a scoping or systematic review or meta-analysis.

We applied the following caveats to our inclusion and exclusion criteria: (1) Employment status—articles needed to specifically assess whether employment was a concern to respondents; reporting employment status or change in employment alone was insufficient for inclusion; (2) Financial hardship—articles needed to include a specific measure of financial hardship or financial toxicity or include self-report of financial difficulty. Description of patient income, treatment cost, or of out-of-pocket expenses alone was insufficient for inclusion, given previous work to measure costs of caregiving [1, 11]. Further, we included studies that assessed financial hardship as part of a larger scale, either as a subscale or an specific item, only if there was specific mention of financial hardship findings in the abstract, as we took this an indication of financial hardship being a primary or secondary focus of the study. In addition, articles focusing on the caregivers of adolescents and young adults with cancer were included only if it was clear that the sample included young adults. Finally, given prior systematic and scoping reviews on the topics, we excluded articles that addressed only caregiver burden, social support, or quality of life, without a specific assessment of a social need [5–9]. 

### Article Review Process

Standard best practices guided our approach to the review [14]. Once the literature search was complete, article titles and abstracts were imported into Covidence software to facilitate the review process. Team members BT and TT provided training on software usage and methods for screening titles and abstracts for initial inclusion. Each abstract was then reviewed independently by two team members, with conflicts resolved through discussion with the senior member of the team (BT, TT) who did not participate in the initial review. The process was repeated for full text articles included after abstract review.

Jointly, the team developed and refined a data extraction template that included details about funding source, research aims, study design, data collection (cross-sectional or longitudinal), theory application (mentioning a theory, conceptual model, or conceptual framework), participant characteristics (both caregivers and patients, if included), sample size, social needs assessed, measurement of social needs, and primary findings. Two members of the study team (SMM, GS) then each independently conducted data extraction for all included articles, with consensus provided as needed by either BT or TT. Senior team members were also available to answer questions or provide clarification as needed. Extracted data were compiled into tables and graphs, and the team then met to discuss results and implications.

## Results

**Fig. 1:**
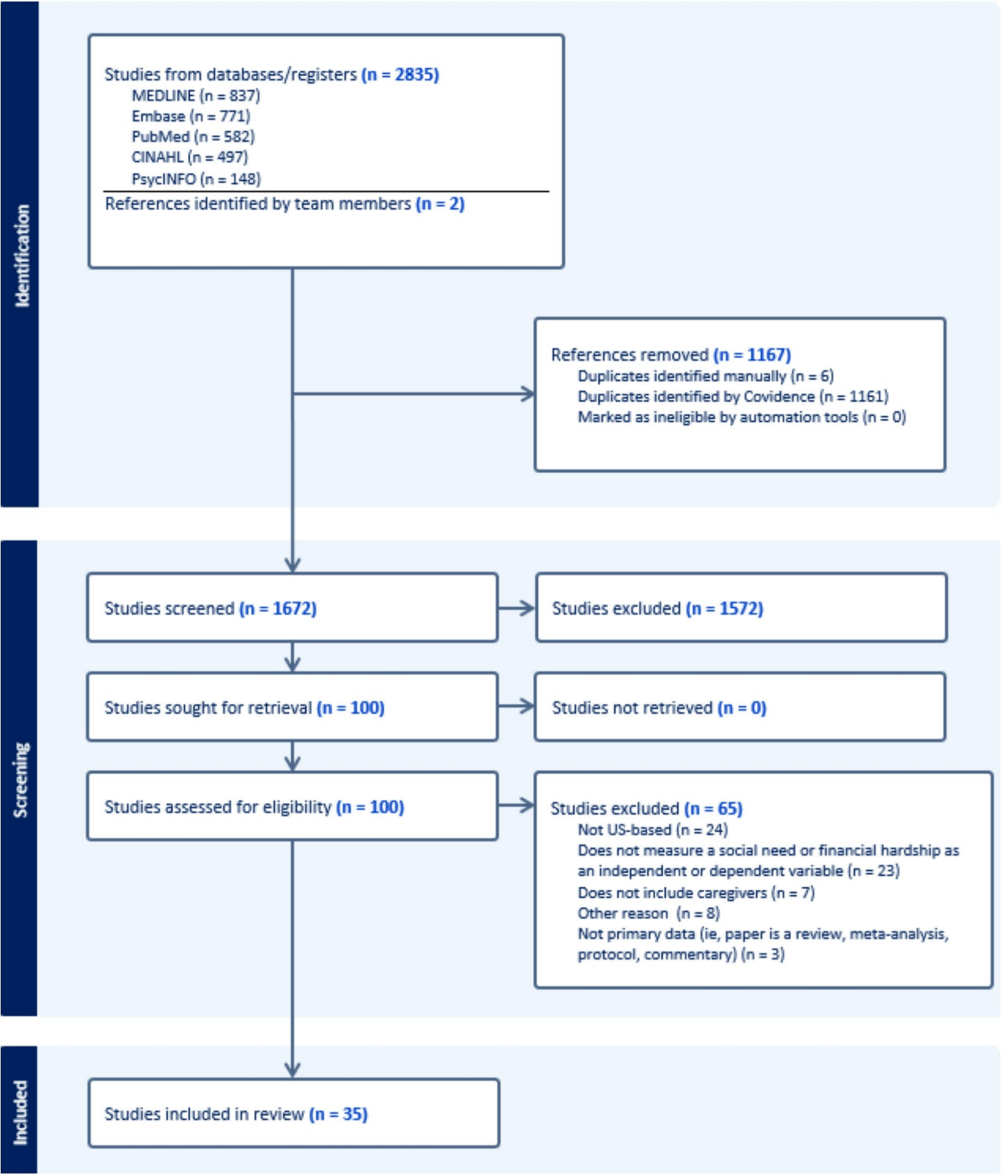
Flow diagram of included records

The initial search yielded 2837 articles, of which 1167 duplicates were eliminated, resulting in 1670 titles/abstracts to screen for inclusion. Post screening,100 articles were included for full-text review. Of these, 35 articles met inclusion criteria. Table 1 provides a complete listing of included studies. Most common reasons for exclusion of full text articles (*n* = 65) were studies based outside the US (*n* = 24), lack of caregivers (*n* = 7), no measure or mention of a social need as a dependent or independent variable (*n* = 23), and lack of primary data (*n* = 3), with eight studies excluded for other reasons (Fig. 1).

**Table 1:** List of articles included in the review (*N* = 35) and their characteristics

Citation	Study aim	General study design	Type of caregiver included	Caregiver sample size	Social need/FH is primary focus	Social needs assessed	Social need measurement	Main caregiver social needs/FH findings
1. Badger et al., 2024 [15]	To determine the relationship between social determinants of health, psychological distress, and caregiver burden among caregivers from three ethnic/racial groups	Cross-sectional survey	Mix of spouses/partners and other caregivers	396	No	Financial/ economic	COST; CRA	Compared to other groups, Hispanic caregivers reported higher caregiver burden related to scheduling, family, and financial concerns. Caregiver burden was associated with being female, Hispanic, married, or socially isolated; having an income that barely or did not meet needs; and lower physical functioning.
2. Baum et al., 2022 [16]	To examine the relationship between mental health (post-traumatic stress symptoms; PTSS) and financial toxicity in adolescent and young adult oncology patients and their caregivers around time of diagnosis	Cross-sectional survey	Mix of spouses/partners and other caregivers	37	Yes	Financial/ economic	COST	PTSS was more common in caregivers than in patients; there was no correlation within the dyads. Median caregiver COST score was 22.0. PTSS was significantly related to financial toxicity in caregivers. Financially was positively correlated among dyad members.
3. Beauchemin et al., 2024 [40]	To describe needs and preferences for dealing with financial toxicity in adolescents/emerging adults with cancer and caregivers	Qualitative	Mix of spouses/partners and other caregivers	7	Yes	Financial/ economic; Transportation; Food; Housing; Utilities	Semi-structured interview	AYA and their caregivers reported challenges and unmet health-related social needs during cancer. Caregivers described cost burdens of transportation and patient medication, and they described ways that community support could help them make ends meet.
4. Dumitra et al., 2018 [27]	To examine the emotional effects of cancer on breast cancer patients and their partners	Cross-sectional survey	Spouses/partners	321	No	Financial/ economic	Investigator –designed Likert-style distress screening	6% of caregivers reported severe/very severe financial concerns, and 13% reported moderate financial concerns. For patients and partners, financial distress was related to higher emotional distress.
5. Edward et al., 2023 [17]	To examine the acceptability, feasibility, and initial effectiveness of an oncology financial navigation intervention for hematologic cancer patients and their caregivers	Feasibility and acceptability trial	Mix of spouses/partners and other caregivers	32	Yes	Financial/ economic	COST; NCCN DT; MEPS-ECSS	The navigation intervention was deemed acceptable. Most (85%) caregivers had baseline COST scores < = 24, and financial toxicity significantly decreased after the intervention.
6. Edward et al., 2024 [18]	To assess the association between financial toxicity and quality of life for hematologic cancer patients going through bone marrow transplant and their caregivers	Longitudinal dyadic survey	Mix of spouses/partners and other caregivers	34	Yes	Financial/ economic	COST; MEPS-ECSS	Patients and caregivers who reported lower financial toxicity had better physical and mental health outcomes when compared to those with more financial toxicity. In addition, caregivers who took time off from work had lower depressive symptoms compared to those whose caregivers did not take time off from work.
7. Fenton et al., 2022 [28]	To assess differences in caregiving responsibilities and social/emotional and financial burdens between adult-child and spousal caregivers	Cross-sectional survey	Mix of spouses/partners and other caregivers	1234	Yes	Financial/ economic	Investigator –designed Likert-style questions	Caregivers who were adult children reported higher financial burden than spouse/partner caregivers, but the difference was not statistically significant. Male caregivers had lower financial burden than female caregivers.
8. Ferrell et al., 2018 [47]	To examine quality of life and financial strain of family caregivers of patients with cancer	Qualitative	Unspecified family caregivers	20	Yes	Financial/ economic	Structured interview	Many caregivers reported financial concerns, and these concerns were associated with emergent social needs.
9. Flora et al., 2023 [22]	To evaluate the caregiver role in treatment decision-making when caring for a patient with classic Hodgkin lymphoma	Cross-sectional survey	Mix of spouses/partners and other caregivers	209	No	Employment concerns	WPAI: CG	Caregiving had an impact on work productivity, regardless of the type of relationship the caregivers had with the patient.
10. Fong et al., 2022 [23]	To examine the role of working for pay among caregivers in the recovery period for patients with pancreatic and periampullary cancer post-pancreatectomy	Cross-sectional survey	Mix of spouses/partners and other caregivers	240	Yes	Employment concerns; Financial/ economic	WPAI: CG; Absenteeism; Presenteeism	Nearly one-quarter of caregivers reported financial difficulties: compared to nonworking caregivers, caregivers who worked had higher financial difficulties (34% of working caregivers vs. 15% of non-working caregivers). Caregivers who used respite care were less likely to report financial difficulty.
11. Ghazal et al., 2023 [46]	To examine the association of financial toxicity and health related quality of life in partners of colorectal cancer survivors	Mixed-methods	Mix of spouses/partners and other caregivers	307	Yes	Financial/ economic	Personal Financial Burden scale; single items to assess debt and financial worry; open-ended narrative question	Long-term financial toxicity was associated with worse quality of life in partners. 63% of partners experienced financial burdens, 29% experienced debt, and 36% reported high financial worry. Younger partners had greater debt and financial burden.
12. Hartnett et al., 2016 [61]	To examine caregiver burden in caregivers of patients with end-stage ovarian cancer and analyze correlates of burden	Cross-sectional survey	Mix of spouses/partners and other caregivers	50	No	Financial/ economic	CRA	Although being a caregiver could be a burden, most caregivers took pride in doing it. Challenges included financial burden and schedule disruption.
13. Hastert et al., 2020 [30]	To understand how caregiving and taking time off from work impacts caregiving, financial burden and psychological outcomes in employed caregivers of African American cancer survivors	Cross-sectional survey	Mix of spouses/partners and other caregivers	202	Yes	Employment concerns; Financial/ economic	Investigator-designed instrument	Employment changes due to caregiving were significantly related to financial burden, and high financial burden was associated with anxiety. 75% of the employed caregivers had to make employment changes or take time off to provide care.
14. Hastert et al., 2024 [29]	To examine correlations between caregiving costs and financial burden in caregivers for African Americans with a history of cancer	Cross-sectional survey	Mix of spouses/partners and other caregivers.	964	Yes	Financial/ economic; Transportation; Food insecurity	Investigator-designed instrument	Nearly two-thirds (64%) of caregivers reported caregiving costs, including transportation (49%) and groceries (33%). 38% of caregivers said their financial resources were not enough to pay for caregiving costs. Financial burden was greater in younger caregivers, those with lower incomes, and those caring for patients diagnosed with regional/distant cancer.
15. Hwang et al., 2003 [25]	To examine caregiver outcomes among individuals providing care for VA Medical Center patients with symptomatic advanced cancer by identifying caregiver characteristics and unmet needs and exploring the association between unmet needs, caregiver burden, and caregiver satisfaction	Cross-sectional survey	Mix of spouses/partners and other caregivers	100	No	Employment concerns; Financial/ economic	Care Strain Index	About one-third of the sample (30%) reported experiencing financial strain; about one-quarter (24%) reported adjustments to work schedule.
16. Jaffe et al., 2021 [62]	To study changes in employment status and coping techniques to manage these changes	Qualitative	Mix of spouses/partners and other caregivers	11	Yes	Employment concerns; Financial/ economic	Semi-structured interview	The qualitative portion of the study found that the three themes that came up among caregivers and cancer survivors were work disruption, work accommodations, and coping mechanisms to address the disruption. They both shared a concern about the lack of support at work with navigating issues caused by the changes in employment.
17. Kavanaugh et al., 2015 [35]	To analyze the correlates of economic burden in spousal caregivers for lung cancer patients	Cross-sectional survey	Spouses/partners	138	Yes	Financial/ economic	Economic burden measure based on the Family Impact Survey	Economic burden was significantly associated with patient symptoms, education, and number of children in the home. There was an indirect path between two factors (age and income) that went through patient symptoms to economic burden. Younger caregivers for patients with greater symptoms reported higher burden than older caregivers.
18. Lee et al., 2022 [43]	To assess risk of cardiometabolic disease due to stress and burden between caregivers and non-caregivers	Cross-sectional survey	Mix of spouses/partners and other caregivers.	83	No	Financial/ economic	CRA	Caregivers of adult cancer patients had a higher risk for cardiometabolic illnesses compared to non-caregivers.
19. Longacre et al., 2021 [36]	To measure cancer caregivers’ emotional, physical, and financial strain during the COVID-19 pandemic	Cross-sectional survey	Mix of spouses/partners and other caregivers	285	Yes	Financial/ economic	Caregiver Quality of Life Index	Financial strain was higher now for non-Hispanic Black caregivers than prior to COVID. Caregivers in the sample were experiencing moderate financial strain.
20. Longacre et al., 2023 [37]	To assess the psychometric properties of a measure of caregiver physical, emotional, and financial strain	Cross-sectional survey	Mix of spouses/partners and other caregivers	299	No	Financial/ economic	National Alliance for Caregiving survey score (NAC-3)	The NAC-3 and its subscales demonstrated moderate to strong association with other validated measures of caregiver strain.
21. Mazanec et al., 2011 [24]	To assess cancer caregivers’ health and work productivity	Cross-sectional survey	Mix of spouses/partners and other caregivers	70	Yes	Employment concerns; Financial/ economic	CRA; WPAI: CG	Caregivers who were employed had to take time off work and had work loss because they had to spend more time being caregivers.
22. McDougall et al., 2022 [41]	To understand food insecurity in caregivers and cancer survivors	Qualitative	Mix of spouses/partners and other caregivers	11	Yes	Food insecurity; Financial/ economic	Semi-structured interviews	Caregivers and patients tried to balance affording medical care with buying healthy food. Multiple caregivers described skipping meals so that the patient could have healthy food. Survivors and caregivers found it acceptable to screen for food insecurity in clinical settings.
23. Milbury et al., 2013 [45]	To examine correlations between caregiving reactions (self-esteem and burden) and distress in dyads (lung cancer patients and spouses)	Cohort study	Spouses/partners	167	No	Financial/ economic	CRA	Spousal report of financial strain at baseline predicted their 6-month distress scores. Spouses reported less financial strain at 3 months and 6 months than they did at baseline.
24. Nguyen et al., 2023 [19]	To describe the impact of financial toxicity in head and neck cancer patients and their caregivers	Cross-sectional survey	Mix of spouses/partners and other caregivers	9	Yes	Financial/ economic	COST	Nearly one-half (44%) of caregivers reported having high financial toxicity, and they reported high concerns about future financial health and their ability to help with the patients care financially.
25. Nightingale et al., 2022 [42]	To describe the impact of financial burden on caregivers of AYA survivors and to understand mechanisms to better provide support for them	Qualitative	Parents and custodial grandparents	24	Yes	Financial/ economic; Transportation; Food; Employment concerns	Semi-structured interviews	The study adapted the conceptual model of financial burden to reflect perspectives of AYAs, oncology providers, and caregivers. The study’s results deepen knowledge of material, psychosocial, and behavioral domains of the adapted patient- and provider-informed conceptual model of cancer-related financial burden, especially highlighting nuanced experiences of caregivers of AYA cancer patients in different phases of the AYA lifespan.
26. Ruff et al., 2024 [63]	To understand how neoadjuvant therapy (NT) affects caregivers of cancer patients with pancreatic ductal adenocarcinoma	Mixed-methods	Mix of spouses/partners and other caregivers	28	No	Financial/ economic	Caregiver Quality of Life Index-Cancer; Semi-structured interviews	Caregivers experienced emotional symptoms that impact their daily lives knowing that their loved ones are undergoing NT. 60% of caregivers shared that it impacted their daily lives that led to decreased work hours that then led to financial challenges.
27. Sadigh et al., 2022 [20]	To explore whether a modified version of the COST was effective to determine factors associated with financial toxicity in patients and caregivers	Cross-sectional survey	Mix of spouses/partners and other caregivers	100	Yes	Financial/ economic	COST	Patients and caregivers experienced significant financial distress, leading to worsened mental health, and reduced quality of life. Findings highlighted the need for financial counseling, policy changes, and support systems to alleviate this burden.
28. Sherwood et al., 2008 [26]	To determine how employment and loss of hours from work impact caregivers of someone with a primary malignant brain tumor.	Cross-sectional survey	Mix of spouses/partners and other caregivers	95	Yes	Employment concerns; Financial/ economic	CRA; lost hours from work due to care demands	Financial burden on the CRA was not significantly associated with current caregiver employment or lost hours from work. Lost hours from work were more commonly reported if the patient was closer to diagnosis or needed assistance with instrumental activities of daily living.
29. Siefert et al., 2008 [64]	To examine the association between social factors and outcomes in caregivers for cancer patients	Cross-sectional survey	Mix of spouses/partners and other caregivers	54	No	Employment concerns; Financial/ economic	CRA	African American and Hispanic family caregivers reported more financial burden due to finances than did white caregivers.
30. Skalla et al., 2013 [39	To identify the physical, psychological, social and spiritual needs of cancer caregivers	Cross-sectional survey	Mix of spouses/partners and other caregivers	172	No	Financial/ economic	Caregiver adaptation of the Cancer Survivor Web-based Needs Assessment Survey (CS-WEBS)	16% of caregivers reported having financial problems, but 3% said they were not bothered by them. Increased debt and loss of assets were the most common financial problems.
31. Tanco et al. 2018 [38]	To analyze the feasibility of using the Edmonton Symptom Assessment System (ESAS) to evaluate caregiver burden	Cross-sectional study	Mix of spouses/partners and other caregivers	90	No	Financial/ economic	ESAS	More than half of caregivers (58%) reported at least mild financial distress; nearly one-third (32%) reported moderate or greater financial distress. Financial distress was significantly correlated (*r* = .34) in patients and caregivers.
32. Tay et al., 2022 [31]	To analyze the relationship between contextual and appraisal factors on hospice family caregivers’ outcomes (wellbeing and mental health)	Cross-sectional survey	Mix of spouses/partners and other caregivers	102	No	Financial/ economic	Investigator-designed item measuring financial adequacy	Perceived financial adequacy was inversely associated with symptoms of depression.
33. Thomas Hebdon et al., 2022 [44]	To analyze associations between caregiving burden for hospice patients and mental health, and determine whether preparedness for caregiving affects those relationships among people who are employed	Cross-sectional survey	Mix of spouses/partners and other caregivers	166	Yes	Employment concerns; Financial/ economic	CRA	There was a significant correlation between financial burden and anxiety. Mediation analyses showed that financial burden was related to anxiety and depression through caregiving preparedness.
34. Van Houtven et al., 2023 [32]	To understand economic cost of a family caregivers for a patient with an advanced cancer	Cohort Study	Type of caregiver not specified	198	Yes	Financial/ economic	One investigator-designed item (“Which one of the following statements best describes your own personal economic situation?”)	Nearly one-quarter of caregivers (24%) reported being in an adverse economic situation. Greater number of caregiving tasks was associated with a greater likelihood of reporting being in an adverse economic situation.
35. Whisenant et al., 2023 [33]	To examine parenting needs and service preferences of advanced cancer patients and their spouses/co-parents of minor children	Mixed-methods	Spouses/partners	21	Yes	Food insecurity Transportation; Employment; Financial/ economic	Investigator-designed survey; Semi-structured interviews	Couples reported needing support for childcare, transportation, meals, home maintenance, and finances.

*Abbreviations: FH * financial hardship, *COST* COmprehensive Score for financial Toxicity, *NCCN DT* National Comprehensive Cancer Network Distress Thermometer and Problem List, *MEPS-ECSS * Medical Expenditure Panel Survey‐ Experiences with Cancer Survivorship Supplement, *CRA* Caregiver Reaction Assessment, *ESAS* Edmonton Symptom Assessment Scale, *WPAI: CG* Work Productivity and Activity Impairment Caregiver

### Study and Sample Attributes

**Fig. 2:**
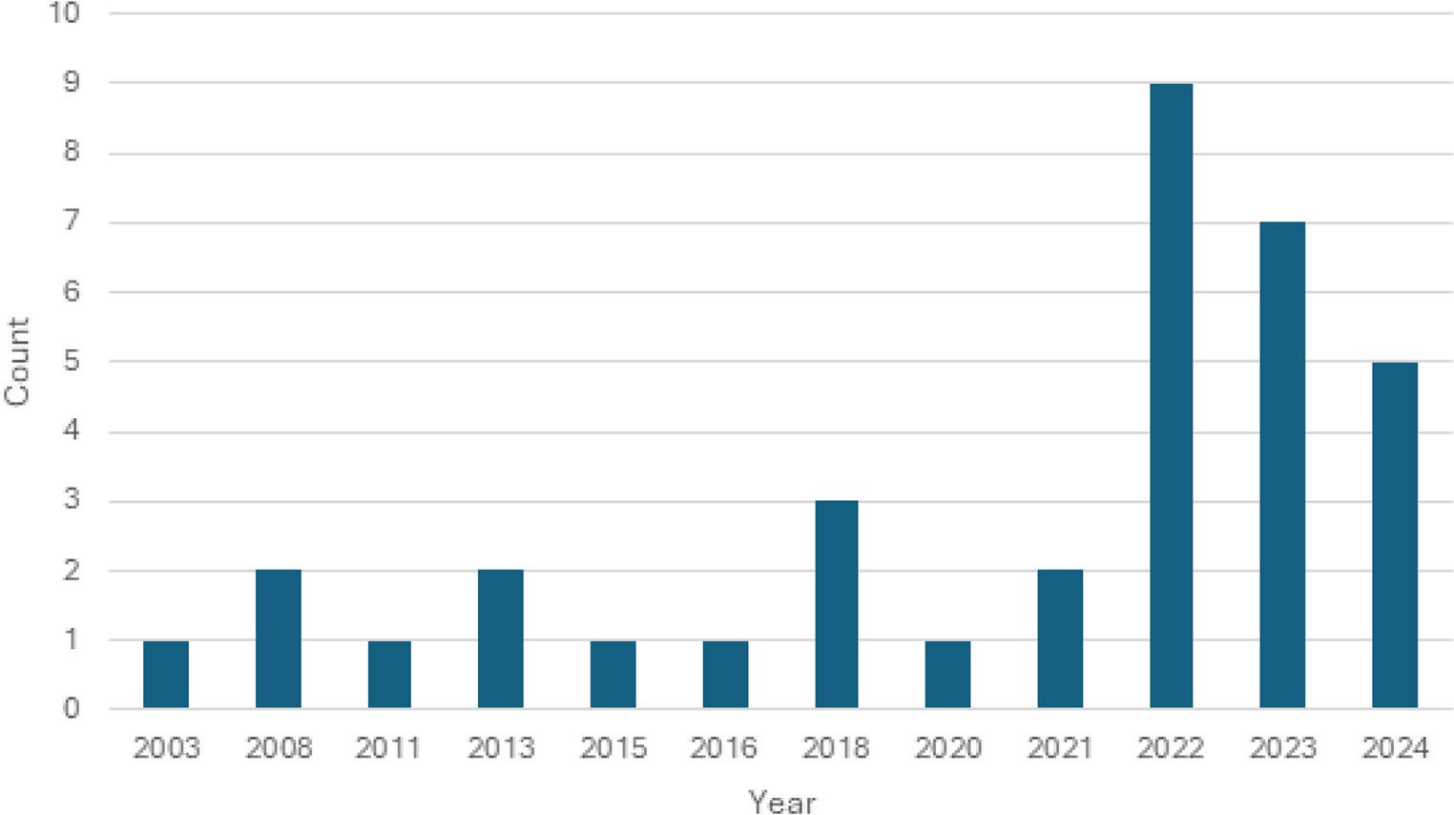
Year of publication of included articles*. *Excluded years indicate no publications

As noted in Fig. 2, publications addressing the social needs of cancer caregivers have increased rapidly in recent years. Across the 25-year time frame of the review period (2000–2024), 60% (*n* = 21) of included articles were published from 2022 to 2024, while most of earlier years saw 0–1 publications. The majority of reviewed articles (77%; *n* = 27) were quantitative, with five qualitative studies and three mixed/multiple-methods studies included as well. Most studies (91%) were cross-sectional in nature, and most (60%) did not report the inclusion of a theoretical or conceptual framework.

**Fig. 3:**
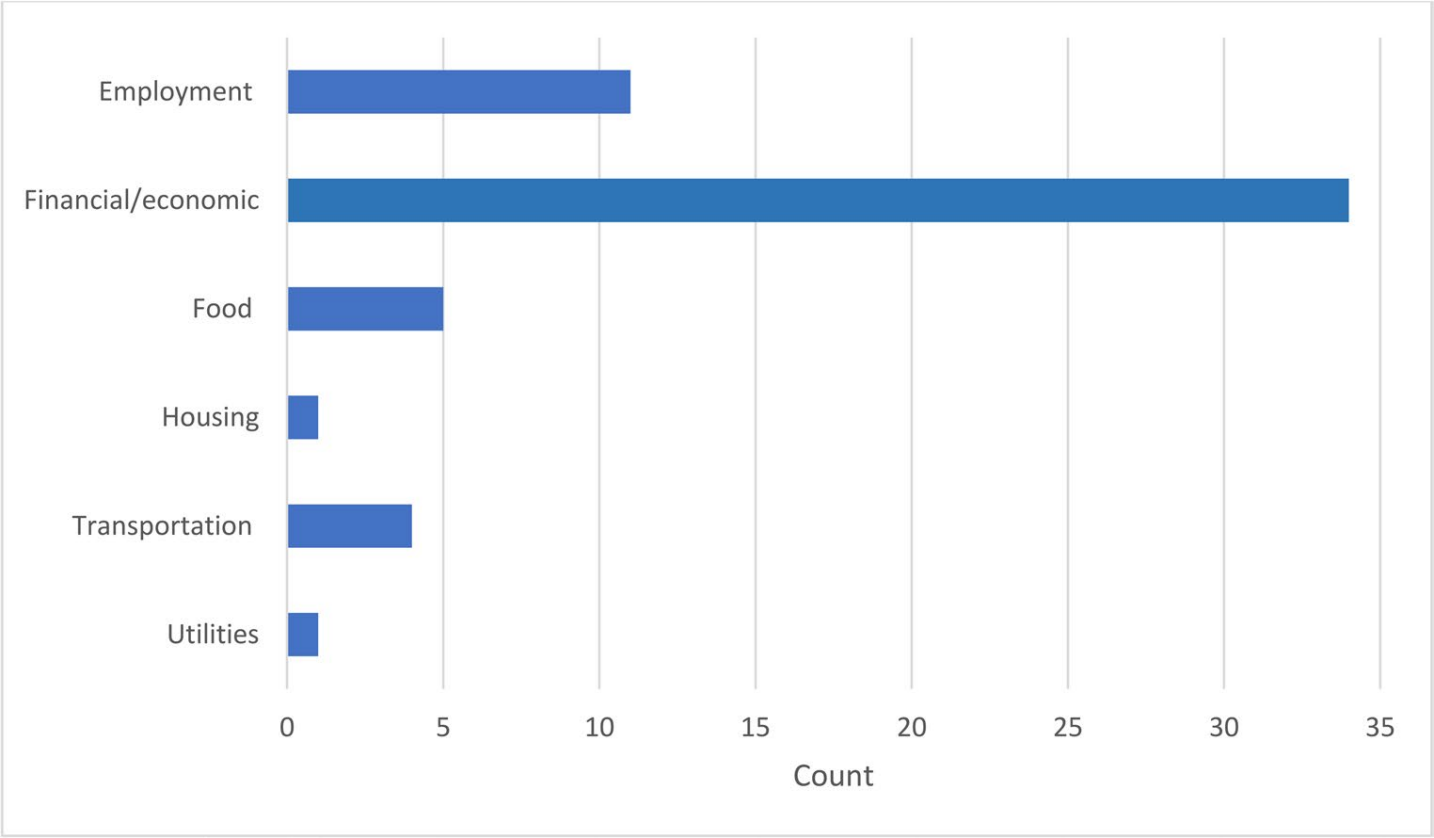
Count of articles addressing financial hardship or social need

Nearly all articles (97%; *n* = 34) addressed some aspect of caregivers’ financial/economic concerns. Other social needs identified included employment concerns (*n* = 11); transportation difficulties (*n* = 4); and food insecurity (*n* = 5). Housing and utilities were covered in one study. Four of the five included qualitative studies reported on at least one social need (Fig. 3).

“Caregivers” were defined broadly across the studies, with 80% (*n* = 26) of studies including a combination of spouses/partners and other caregivers in their samples. Other types of caregivers included friends, parents, grandparents, and unspecified family caregivers. Four studies included only spouses/caregivers. Just over one-half of the studies (54%, *n* = 19) included both patients and caregivers, with five quantitative studies applying dyadic analysis techniques; the remainder of articles were focused on caregivers only. Sample sizes of caregivers ranged from 7 to 1234, reflective of the variation in study design. Of studies that included a breakdown of gender among their samples (*n* = 32), 91% (*n* = 29) were majority female. Due to variations in reporting of race and ethnicity (i.e., some studies reported combined race and ethnicity, while others reported race and ethnicity separately), we were unable to compare racial and ethnic compositions across samples. Similarly, variation in the reporting of age (median, mean, range) prohibited us from assessing ages of respondents across studies.

### Measurement and Assessment of Social Needs

Broadly, there was little consistency around social need measurement across the included quantitative and mixed/multiple methods studies. The Comprehensive Score for Financial Toxicity (COST) assessed financial hardship in six of the studies [15–20]. This 11- or 12-item patient-reported outcome scale has become a widely used tool in research to capture financial toxicity in the cancer context [21]. The Sadigh et al. (2022) study specifically explored the psychometric properties of a version of the COST adapted for caregivers, finding high internal consistency and convergent validity with the patient version [20]. 

Three of the studies that assessed employment issues among caregivers used the Work Productivity and Activity Impairment Caregiver (WPAI: CG) scale [22–24]. Other mechanisms to capture employment issues included assessments of presenteeism and absenteeism, the Care Strain Index, and self-report of lost work hours due to caregiving [23, 25, 26]. Seven studies, meanwhile, used investigator-designed surveys that consisted of a wide variety of questions, such as Likert-style agreement rankings and subsets of other validated scales, including questions drawn or adapted from the Medical Expenditure Panel Survey [27–33]. 

Several articles measured social needs peripherally as one component of a larger scale. For example, our search terms and strategy resulted in the inclusion of several articles (*n* = 8) that used the Caregiver Reaction Assessment (CRA), which captures financial needs as a component of caregiver burden [34]. Other broader measures of caregiver burden and quality of life that were included in studies meeting inclusion criteria were the Family Index Survey, the Care Strain Index, the Caregiver Quality of Life Index [25, 35, 36]. Of note, three studies were evaluations of newly created or adapted scales to assess caregiver burden, of which financial concerns were identified as components of burden [37–39]. 

Among qualitative studies and the qualitative components of multiple/mixed methods studies, data collection methods included semi-structured interviews (*n* = 6), structured interviews (*n* = 1), and open-ended narrative questions (*n* = 1). Qualitative methods were used in the majority of studies related to food (4 out of 5 studies addressing food insecurity had a qualitative component) [33, 40–42] and transportation (3 out of 4 studies addressing transportation difficulties had a qualitative component) [33, 40, 42] needs. Housing and utilities concerns were also addressed through qualitative inquiry [40]. 

### Associations with Physical and Psychosocial Outcomes

Twenty of the 35 studies (57%) attempted to assess associations between unmet social needs and physical and/or psychosocial outcomes, including quality of life. Lee et al. (2021) explicitly addressed caregiver physical health through blood assays, finding elevated cardiometabolic risk among cancer caregivers, which the authors suggest may be driven by the high levels of stress caregivers face [43]. A variety of psychosocial and mental health outcomes, including post-traumatic stress, distress, anxiety, depression, were also explored in the reviewed studies, with more needs universally correlating with higher symptomology [15, 16, 27, 30, 31, 44, 45]. 

To explore associations between financial hardship and health-related quality of life, Edward et al. (2024) and Ghazal et al. (2023) used the versions of the Patient-Reported Outcomes Measurement Information System Profile (PROMIS), which included subscale measures of physical (e.g., sleep, pain, physical functioning) and mental (e.g., anxiety, depression) health, with both studies finding inverse associations between social needs and health outcomes, such that more financial hardship (or elements of hardship, such as medical debt or financial worry) was correlated with worse health [18, 46]. Quality of life and overall well-being were also explored in relation to social needs in three other reviewed studies, and similar associations were found between more unmet social needs (in these cases, financial hardship) and worse outcomes [20, 31, 47]. 

### Interventions to Address Unmet Social Needs/Financial Hardship

Only one study (Edward et al., 2023) described an intervention, a financial navigation program for patients with hematologic cancer and their caregivers [17]. In this pilot study, both patients who screened positive for financial hardship and the caregivers of patients undergoing a bone marrow transplant were recruited to participate in the navigation intervention. The navigation intervention demonstrated preliminary efficacy in reducing financial hardship in caregivers, and patients and caregivers found the intervention to be acceptable and feasible.

While not an intervention itself, a qualitative study by Ferrell et al. (2018), was conducted in association with a randomized controlled trial of a support intervention in 225 family caregivers [28]. The authors noted that over the course of the first year of the trial, the research team identified a need to qualitatively explore the financial concerns emerging among caregivers. Beauchemin et al. (2024), meanwhile, used qualitative methods to characterize patient and caregiver perspectives on intervention development related to financial hardship screening among caregivers of AYA patients, finding that caregivers favored clear and consistent approaches in the clinical setting to alleviate financial burdens. Other included qualitative work also took steps toward intervention development through inquiry around perceptions of needed support, views on in-clinic screenings for social needs, and preference for support service delivery [33, 41, 42]. 

## Discussion

The findings from this scoping review highlight the varied landscape of assessment of social needs among cancer caregivers. As in our scoping review of social needs in patients with cancer, the measurement and assessment of financial concerns and hardship in caregivers far exceed that of other social needs [1]. Employment concerns, which closely link to financial concerns, were assessed in nearly one-third of studies, but basic social needs, such as food, transportation, housing, and utilities, have been examined relatively infrequently among cancer caregivers.

Our findings on the high volume of financial hardship research in caregivers reflect broader trends in the growing focus on financial toxicity over the past decade [48]. Studies in this regard first named the problem and then focused on describing its predictors and consequences—initially generally and then in specific populations, disease types, and, as found in this review, cancer caregivers. More recently, research has shifted to intervention development and pilot testing, with the first large trials of interventions in patients still emerging [49–52]. Given the frequency of financial concerns demonstrated among the studies included in this review, future research toward interventions for caregivers, either alone or as part of a dyad, are needed.

Caregiving clinics have emerged as a resource for cancer caregivers and may offer an opportunity to screen for unmet social needs, provide targeted interventions, and connect those at risk with appropriate support services and resources [53]. A recent survey of 238 Commission on Cancer-accredited cancer centers in the United States found that 75% had at least one program specifically targeted to family caregivers [54]. In addition, oncology navigation services, including financial navigation, may help patients optimize offered services and connect them to available resources—in the cases where the social needs of patients and caregivers overlap, including caregivers in navigation may help to mitigate their unmet needs [55, 56]. 

Several studies included in the review measured financial hardship as a subscale of other measures of caregiver burden (e.g., CRA, NAC-3). The CRA financial subscale, which is one of five subscales (with self-esteem, family support, schedules, and health as the others), includes three questions that address elements of financial strain, including decreased income, increased expenses, and cost-coping (i.e., reducing spending in other areas because of caregiving-related expenses). The CRA also briefly addresses employment through one question in its “Disrupted Schedules” subscale (“Have you had to cut back on work hours or change your work schedule?”). Likewise, the National Comprehensive Cancer Network (NCCN) Distress Thermometer and Problem List includes a “Practical Problems” checklist where respondents can report transportation, employment, financial, and housing concerns [17]. To that end, these tools may enable the clinical team in caregiving clinics to succinctly and efficiently assess the complex, co-occurring challenges caregivers face, both related to unmet social needs and other dimensions of caregiver burden.

It is important to note that financial hardship can both precede and result from cancer treatment, and the measures included in the reviewed articles did not typically distinguish the timing of the reported financial hardship. This speaks to an opportunity for clinical teams in caregiving clinics to screen upon clinic entry and throughout the patient’s treatment trajectory to identify potential risk at the outset of treatment and as medical bills and non-medical costs accumulate over time during treatment. Abbreviated versions of the COST or newly-developed measures that are shorter in length may facilitate these efforts in the clinical setting [57–59]. 

In addition to suggesting areas for clinical impact, the gaps in the literature identified in our review point to important directions for future research. Firstly, given the limited focus on unmet social needs beyond financial and employment concerns, there is a significant opportunity for research teams to explore a broader range of social needs affecting caregivers. The qualitative studies within our review shed light on the interconnected nature of these needs, providing a foundation for future work to expand upon through quantitative inquiry in larger samples. Secondly, nearly all of the studies included in the review were cross-sectional in nature, highlighting the need for longitudinal assessments of caregivers’ unmet social needs as patients move through active treatment and into longer-term survivorship. This is of particular importance in light of cumulative effects of missed work and high cost sharing/out-of-pocket expenses throughout the course of the patient’s treatment and the caregiving experience, as well as evidence that social needs may shift over time [10, 11, 60]. Thirdly, as demonstrated by the inclusion of only one study focused on an intervention, future efforts must move beyond merely describing caregivers’ unmet social needs and work towards developing and testing strategies to address them. Finally, only a limited number of studies in the review applied dyadic analysis techniques. These approaches allow research teams to consider the interdependence between patient and caregiver responses: that is, how one respondent’s experiences impact the other respondent’s responses (and vice versa). In the context of unmet social needs, these approaches may be of particular value among caregiver-patient dyads who cohabitate and/or share resources. The Dyadic Cancer Outcomes Framework offers researchers a useful guide to considering and structuring analytic approaches across the cancer treatment trajectory [12]. 

### Limitations

Findings from this scoping review must be considered within the context of several limitations. Despite our use of expansive terms related to our topics of interest, we may have inadvertently missed articles that used other words or phrases to capture social needs, caregiving, or cancer. To attempt to mitigate this risk, we reviewed references lists among included articles. As noted in our methodology, if an article measured financial hardship or social needs as a subscale of a larger scale (e.g., CRA), we only included it in the review if it reported related findings in the abstract; it is possible we may have excluded articles that described relevant findings in the body of the manuscript. Due to the distinctive, fragmented nature of the US healthcare system and its growing reliance on informal caregivers, we limited our review to US based studies, but this choice limits our understanding of caregiving experiences to those within the US.

## Conclusions

This scoping review of 35 studies published in the past 25 years provides important preliminary evidence about unmet social needs and financial hardship among cancer caregivers. Consistent with previous work in patients with cancer, the included studies reported connections between financial hardship/needs and quality of life-related outcomes in caregivers. At the same time, we have identified several key areas for future research. To support caregivers and improve outcomes for both patients and caregivers, it will be important to understand how multiple needs interact and evolve over time and test effective strategies to address them.

## Key References

- Thompson T, Doherty M, Berrett-Abebe J, et al. Social Needs in Cancer Survivors: A Scoping Review and Future Directions. *Curr Oncol Rep*. May 5 2025. 10.1007/s11912-025-01664-4.
  - This is a comprehensive scoping review of the social needs of cancer survivors.
- Edward JS, McLouth LE, Rayens MK, Eisele LP, Davis TS, Hildebrandt G. Coverage and Cost-of-Care Links: Addressing Financial Toxicity Among Patients With Hematologic Cancer and Their Caregivers. *JCO Oncol Pract*. 2023;19(5):e696-e705. 10.1200/OP.22.00665.
  - This is one of the only interventions, to our knowledge, to address social needs among cancer caregivers.
- Hastert TA, Kyko JM, Ruterbusch JJ, et al. Caregiver costs and financial burden in caregivers of African American cancer survivors. *Journal of cancer survivorship : research and practice*. 2024;18(2):565-574. 10.1007/s11764-022-01271-3.
  - This study focuses on the experiences of African American caregivers, a group frequently under-represented in research.
- Van Houtven CH, Miller KEM, James HJ, et al. Economic costs of family caregiving for persons with advanced stage cancer: a longitudinal cohort study. *J Cancer Surviv*. 2023. 10.1007/s11764-023-01462-6.
  - This study is one of few that takes longitudinal approach to understanding financial hardship and economic concerns among cancer caregivers.
- Beauchemin MP, Solomon S, Michaels CL, et al. Toward identification and intervention to address financial toxicity and unmet health-related social needs among adolescents and emerging adults with cancer and their caregivers: A cross-cultural perspective. *Cancer medicine*. 2024;13(8):e7197. 10.1002/cam4.7197.
  - This qualitative study considers the interaction between social needs and financial hardship and takes steps toward understanding patient and caregiver perspectives for intervention.
- Odom JN, Applebaum A, Bakitas MA, et al. Availability of Family Caregiver Programs in US Cancer Centers. *JAMA Network Open*. 2023;6(10):e2337250-e2337250. 10.1001/jamanetworkopen.2023.37250.
  - This analysis describes the role and availability of caregiving clinics across practice settings. Caregiving clinics offer an opportunity for providers to identify and address the social needs and financial hardship experienced by cancer caregivers.

## Data Availability

No datasets were generated or analysed during the current study.
